# Plant-made vaccines against viral diseases in humans and farm animals

**DOI:** 10.3389/fpls.2023.1170815

**Published:** 2023-03-28

**Authors:** Hang Su, André van Eerde, Espen Rimstad, Ralph Bock, Norica Branza-Nichita, Igor A. Yakovlev, Jihong Liu Clarke

**Affiliations:** ^1^ Division of Biotechnology and Plant Health, NIBIO - Norwegian Institute of Bioeconomy Research, Ås, Norway; ^2^ Faculty of Veterinary Medicine, Norwegian University of Life Sciences, Oslo, Norway; ^3^ Department III, Max Planck Institute of Molecular Plant Physiology, Potsdam-Golm, Germany; ^4^ Department of Viral Glycoproteins, Institute of Biochemistry of the Romanian Academy, Bucharest, Romania

**Keywords:** plant-made vaccines, viral diseases, humans, farm animals, molecular farming, plant transformation

## Abstract

Plants provide not only food and feed, but also herbal medicines and various raw materials for industry. Moreover, plants can be green factories producing high value bioproducts such as biopharmaceuticals and vaccines. Advantages of plant-based production platforms include easy scale-up, cost effectiveness, and high safety as plants are not hosts for human and animal pathogens. Plant cells perform many post-translational modifications that are present in humans and animals and can be essential for biological activity of produced recombinant proteins. Stimulated by progress in plant transformation technologies, substantial efforts have been made in both the public and the private sectors to develop plant-based vaccine production platforms. Recent promising examples include plant-made vaccines against COVID-19 and Ebola. The COVIFENZ^®^ COVID-19 vaccine produced in *Nicotiana benthamiana* has been approved in Canada, and several plant-made influenza vaccines have undergone clinical trials. In this review, we discuss the status of vaccine production in plants and the state of the art in downstream processing according to good manufacturing practice (GMP). We discuss different production approaches, including stable transgenic plants and transient expression technologies, and review selected applications in the area of human and veterinary vaccines. We also highlight specific challenges associated with viral vaccine production for different target organisms, including lower vertebrates (e.g., farmed fish), and discuss future perspectives for the field.

## Introduction

With global climate change, the risk for faster and wider geographical spread of infectious diseases in both human and animals is increasing. Vaccination has proven to be a highly effective measure for protecting humans and farm animals from infectious diseases. In general, vaccines are tightly controlled and thus considered as safe. In addition, they have minimal ecological impact, and are vital for human health and for the sustainability and expansion of global livestock production by providing efficient viral disease prophylaxis and disease control. Recombinant subunit vaccines offer particularly high safety as they cannot revert to virulent agents. They also offer high stability, are often easy to produce, and can have similarly high immunogenicity as full-fledged viral particles (Su and Su, 2018). Antigens, such as viral capsid and envelope proteins, are the key components of recombinant subunit vaccines, and can be expressed in various expression systems such as bacteria, yeast, transiently transformed plants and stable transgenic plants, insects, mammalian cell cultures, and cell-free systems. From these platforms, they can be isolated and formulated as vaccines.

With the continuing advancement of genetic engineering technologies, plants are increasingly used as bio-factories for the expression of antibodies as well as foreign antigens. Plants are advantageous as bio-factories, because they are inexpensive to grow at a large scale in greenhouses, bioreactors or open fields. Plants can express complex antigens while avoiding the risk of carrying human pathogens or endotoxins, which can contaminate viral, bacterial, and insect or mammalian cell expression systems (Aggarwal, 2009). In the present review, we discuss the status of vaccine production in plants using selected applications in human and veterinary vaccines as examples. We also highlight specific challenges associated with viral vaccine production for different target organisms, including lower vertebrates (e.g., farmed fish), and discuss future perspectives for the field. We argue that plant-based vaccines offer great promise in combating a range of viral diseases and can be a major avenue for low-cost and easily administered vaccines in the future.

## Plant expression platform for vaccine preparation

Foreign proteins, including antigens, can be produced transiently in plants or stably in genetically modified (GM) plants. The underlying technologies have been established as an alternative option to mainstream production systems in microbes or animal cell cultures (Clarke et al., 2013; Bock, 2015; Dobrica et al., 2017; Castells-Graells and Lomonossoff, 2021). The feasibility, efficacy and safety of plant-based production of human and animal vaccines have been demonstrated by many successful examples and in numerous clinical studies (Takeyama et al., 2015; reviewed, e.g., in LeBlanc et al., 2020; Stander et al., 2022; Zahmanova et al., 2022). The strategy for vaccine production in plants is usually to test different expression vectors carrying the antigen-encoding foreign sequence. Common optimization steps include the test of different promoters, codon optimization of the transgene, test of different leader sequences to enhance translational efficiency, and targeting of the protein product to different subcellular locations (e.g., organelles) by appropriate transit or signal peptides (Joung et al., 2016).

Plant expression platforms provide a number of distinct advantages for the synthesis of recombinant subunit vaccines. Plant-made vaccines can substantially decrease the production costs and the energy input compared to other systems. Plants utilize solar energy and capture CO_2_, making them an environment friendly production system for recombinant proteins (Avesani et al., 2013). The low capital costs also make it easy to scale up plant biomass production for industrial utilization (Abiri et al., 2016). The post-translational modifications, folding and assembly of proteins produced in plant expression systems are often comparable to those in mammalian cells, which is often critical for immunogenicity (Margolin et al., 2020; Margolin et al., 2023). However, there are also some plant-specific features (e.g., in the protein glycosylation patterns) that need to be considered.

Many viral capsid proteins expressed or co-expressed in plants can self-assemble into viral-like particles (VLPs). VLPs don’t include nucleic acid, but have a similar structure, size and repetitive geometry as the wild-type virus, and therefore are ideal for inducing potent host immune responses (Marsian et al., 2017; Nooraei et al., 2021). Plant-made VLPs avoid the risk of insertion of viral nucleic acid sequences into the human genome and the associated potential oncogenesis (e.g., of DNA vaccines), do not trigger undesirable host responses to viral vector-derived vaccines, and do not have the danger of incomplete inactivation of attenuated viruses (Yang et al., 2017). As a subtype of VLP, recombinant plant virus particles (rPVPs) and genetically modified plant virus are increasingly studied as vectors for candidate vaccines (Lebel et al., 2015; Karpova and Nikitin, 2022). Many plant viruses such as the tobacco mosaic virus (TMV), cowpea mosaic virus (CPMV), potato virus X (PVX), alfalfa mosaic virus (AlMV), and papaya mosaic virus (PapMV) are currently used for the development of new vaccines. Specific modification of plant viruses allows dense expression of fused antigens, thereby contributing to the development of an effective immune response (Monreal-Escalante et al., 2022).

Plant-based expression platforms are roughly divided into three categories: transient expression systems, stable expression in the nuclear genome of transgenic plants or cell cultures, and stable expression in the plastid (chloroplast) genome of so-called transplastomic plants (Bock and Warzecha, 2010; Loüssl and Waheed, 2011; Clarke et al., 2013; Monreal-Escalante et al., 2022). Vaccines against viruses have been produced with all three platforms, which each have their specific advantages and limitations (Guan et al., 2013; He W. et al., 2021; Lobato Gómez et al., 2021). Plastid genome engineering in lettuce, tomato, potato, cabbage, and some other edible plants has been developed for recombinant protein production (Zhou et al., 2006; Cardi et al., 2010). The chloroplast expression system has also shown promising potential for oral vaccines (Davoodi-Semiromi et al., 2010). In particular, lettuce chloroplasts have been reported to express high levels of antigens for oral therapy in humans (Lakshmi et al., 2013; van Eerde et al., 2019).

Plant-based vaccines are especially suitable for oral immunization. Edible plants provide the advantage of convenient, needleless administration (Tacket, 2009). Orally administered vaccines can induce the host mucosal and systemic immunity after uptake by M cells in the follicle-associated epithelium in the gastrointestinal tract (Tatsuhiko et al., 2014). Orally delivered plant-produced vaccines can reduce the costs and the time involved in the purification of the antigens, by virtue of the consumed plant biomasses directly inducing immunity. Edible plant material can also be easily freeze-dried for long-term storage at low cost (Su et al., 2015; Rosales-Mendoza, 2020). When ingested as part of the diet, the plant-produced vaccines are protected from the acid environment of the stomach by the plant cell wall and are then slowly released in the gut. Therefore, cold chain facilities are not needed to stock the respective plant material, making plant vaccines more cost efficient and easier to distribute than conventional vaccines. Immune tolerance is considered as a potential barrier to the development of edible vaccines, because high-dosage oral administration can result in tolerance of antigen by the immune system. Therefore, the optimum antigen dosage needs to be carefully determined, and antigen purity and batch-to-batch consistency need to be verified (Jain et al., 2021).

## Plant-made vaccines against viral diseases in humans

### Coronaviruses

Coronaviruses (CoVs), belonging to the family *Coronaviridae*, cause several respiratory diseases in humans and have become intensively studied since their unprecedented spread in the early 21^st^ century (Song et al., 2019). Severe acute respiratory syndrome coronavirus (SARS-CoV) and Middle East respiratory syndrome coronavirus (MERS-CoV) are highly pathogenic beta-coronaviruses (β-CoVs) that pose a substantial threat to public health (Hahn et al., 2007). CoVs infect the human gastrointestinal, respiratory, hepatic and central nervous systems and also can infect a wide range of animals, including farm animals and wild birds, rodents, bats and others (Chen and Guo, 2016). Severe acute respiratory syndrome coronavirus 2 (SARS-CoV-2) emerged in December 2019, and since then, more than 659 million confirmed cases and over 6.6 million deaths have been reported up to January 11, 2023 (https://www.who.int/publications/m/item/weekly-epidemiological-update-on-covid-19—11-january-2023) (Rosales-Mendoza, 2020). Vaccination is one of the most effective strategies among all proposed therapeutic and prevention options for the control of the SARS-CoV-2 pandemic (LiDe and Clercq, 2020). SARS-CoV-2 is an enveloped beta-coronavirus with a positive-sense, single-stranded linear RNA genome, which is large at a size of 26-32 kb (Armbruster et al., 2019). In addition to envelope and capsid proteins, several non-structural proteins such as 3-chymotrypsin-like protease, papain-like protease, helicase, and RNA-dependent RNA polymerase have been considered as attractive targets for vaccine design and development of antiviral agents against CoVs (Li et al., 2020; Malik et al., 2020).

The spike glycoprotein (S) is most appealing because of its placement on the virion surface in the form of homotrimers that take the shape of the typical crown-like appearance of coronaviruses. The spike plays a crucial role in the interaction between the virion and the host cells during entry of viral particles into host cells, and is the major antigen for triggering host protective immune responses (Mahmood et al., 2021). Several vaccines have been developed expressing the S protein and have received local authorization (Funk and Ardakani, 2021). The U.S. Food and Drug Administration (FDA) has approved chewing gum containing clinical-grade plant material of the virus-trapping proteins CTB-ACE2 (angiotensin converting enzyme 2 fused to the non-toxic cholera toxin subunit B, CTB) expressed in chloroplasts of lettuce. Chewing gum with CTB-ACE2 provides an option to protect patients from infection in the oral cavity (Daniell et al., 2022).

New SARS-CoV-2 variants increase the demand for new vaccine expression systems that offer speedy and low-cost production to ensure rapid vaccine availability, especially in developing countries (Fuenmayor and Cervera, 2017; Williams and Burgers, 2021). There have been many successful cases of plant-expressed vaccines and pharmaceuticals developed and evaluated in clinical trials. A SARS-CoV-2 VLP was developed by Medicago (**Table 1**). Its design followed the well-established plant-based production of influenza virus proteins by introducing the corresponding genes into *Nicotiana benthamiana via* Agrobacterium-mediated transformation, leading to the production of influenza VLPs that were similar to the structure of native virions (Ward et al., 2014). Taking a similar approach, the gene for the S protein of SARS-CoV-2 was produced and the resulting candidate vaccine was successful in phase I human clinical trials and is currently undergoing phase 2 and 3 clinical trials (Rosales-Mendoza, 2020; Ward et al., 2021). There have been many attempts to produce SARS-CoV-2 vaccines in plant systems, and they are currently in different phases of clinical trials (**Table 1**). A plant-made SARS-CoV-2 vaccine now seems to be within reach (**Table 1**).

**Table 1:** Examples of plant-based vaccines against SARS-CoV-2.

Antigen/product	Plant	Status	Reference/website
SARS-CoV-2 VLP Vaccine (IBIO-200)	*N. benthamiana*	Pre-clinical	https://www.ibioinc.com/ Maharjan and Choe, 2021; Ortega-Berlanga and Pniewski, 2022
SARS-CoV-2 Subunit Vaccine (IBIO-201)	*N. benthamiana*	Pre-clinical	https://www.ibioinc.com/ Maharjan and Choe, 2021; Ortega-Berlanga and Pniewski, 2022
SARS-CoV-2 Spike protein	*N. benthamiana*	Phase II/III NCT04636697	Gobeil et al., 2021; https://clinicaltrials.gov/ct2/show/NCT04636697
SARS-CoV-2 Subunit Vaccine (KBP-201)	*N. benthamiana*	Phase I/II NCT04473690	https://clinicaltrials.gov/ct2/show/NCT04473690
SARS-CoV-2 VLP Vaccine (CoVLP)	*N. benthamiana*	Phase III NCT05040789	https://clinicaltrials.gov/ct2/show/NCT05040789
SARS-CoV-2-RBD-Fc (Baiya SARS-CoV-2 Vax 2)	*N. benthamiana*	Phase I NCT05197712	https://clinicaltrials.gov/ct2/show/NCT05197712
SARS-CoV-2 N protein	*N. benthamiana*	Commercialized for research/diagnostic use only	https://www.leafexpressionsystems.com/
PtX™ SARS-CoV-2 Spike Protein S1 (His) Ultra	*N. benthamiana*	Commercialized for research/diagnostic use only	https://www.capebiopharms.com/products ptx-spike-protein-s1-his-ultra/

### Hepatitis B and C viruses (HBV and HCV)

More than 350 million people are currently living with chronic HBV or HCV infections that can progress to liver fibrosis, cirrhosis and eventually hepatocellular carcinoma (HCC), the most prevalent form of liver cancer, with a poor survival rate (Llovet et al., 2021). HBV infection is not a curable disease today; current treatment relies on a combination of immunomodulators and direct acting antivirals (DAA), which significantly inhibit virus replication, but have no impact on the covalently closed circular DNA (cccDNA) pool responsible for HBV chronicity (Nassal, 2015). The recent development of orally administered antivirals that are able to cure the HCV infection in more than 90% of treated patients represents a significant breakthrough in HCV therapy. However, most patients in low/medium-income countries have little access to diagnosis and treatment (Quaranta et al, 2021). Prevention of the viral infection remains the most efficient way to reduce HBV/HCV-related morbidity and mortality. According to the World Health Organization (WHO), vaccination could avoid 4.5 million premature deaths caused by HBV/HCV-induced liver diseases by 2030 (WHO, 2022). This assessment urges development of a prophylactic HCV vaccine and improvement of currently available HBV vaccines to address non-responders.

The first attempt to produce a vaccine in plants was the production of HBV small (S) protein antigen (S-HBsAg) in *Nicotiana tabaccum* (Mason et al., 1992). The HBV small (S) envelope protein was the ideal model antigen to test the feasibility of using plants as a production platform for VLPs. Similar to the natural infection, when expressed in heterologous systems, the S protein retains the ability to self-assemble through complex disulfide linkages into spherical, 22-nm subviral particles that are secreted from cells (Eddleston, 1990). These complexes, known as the S-HBsAg, are highly immunogenic and the main component of the commercial HBV subunit vaccine produced in yeast (McAleer et al., 1984). The plant-made protein had similar physical properties to those of the antigen isolated from infected patients and was as immunogenic as the yeast-derived counterpart, confirming the suitability of plant cells for expression of functional, complex antigens (Thanavala et al., 1995). These seminal discoveries have attracted huge scientific interest, including the exploration of other plant species for the production of S-HBsAg, especially edible plants for potential oral administration (reviewed in Pantazica et al., 2021), and efforts to increase the repertoire of HBV-derived antigens in order to enhance the immune response. Consequently, successful production of the S-HBsAg was reported in a variety of plant species, such as potato (Ehsani et al., 1997; Richter et al., 2000);, lettuce (Pniewski et al., 2011), carrot (Imani et al., 2002), peanut (Chen et al., 2002), tomato (Gao et al., 2003; Srinivas et al., 2008), banana (Sunil Kumar et al., 2005), lupin (Pniewski et al., 2006), soybean (Ganapathi et al., 2007) and maize (Hayden et al., 2012), either in whole plants or cell cultures. The antigen expression levels varied significantly between hosts, ranging from a few nanograms to tens of micrograms per gram fresh weight of plant material, with higher yields obtained in transiently transformed plants and transgenic cell cultures (Joung et al., 2016).

In-depth characterization of the biochemical, antigenic and immunogenic properties of the S-HBsAg was performed in most of these preclinical studies, revealing appropriate folding and VLP formation, and induction of protective titers of anti-S antibodies (i.e., ≥10 mIU/mL after the last vaccination dose) (McMahon et al., 2009), depending on the quantity and purity of the administered antigen (Pniewski, 2013; Joung et al., 2016). The ability of the triggered antibodies to neutralize HBV infection *in vitro*, ultimately confirmed the quality of this immune response (Dobrica et al., 2017).

The great variation of the immunization approaches included oral, parenteral or mixed antigen administration regimes, and it appears that parenteral administration has the most encouraging prospects for vaccine development of plant-produced antigens, provided robust and sustainable production together with clinical-grade protein purification are achieved. Successful clinical trials, demonstrating high immunogenicity of M (middle) & L (large) proteins in non-responders to the standard vaccine, have led to the recent FDA approval of a three-antigen HBV vaccine containing the S, M and L proteins produced in mammalian cells (Vesikari et al., 2021). Owing to better immunogenic properties, expression of the two HBV envelope proteins M-HBsAg and L-HBsAg has also been attempted in several plants such as potato, tomato, and *N. tabacum*; however, antigen accumulation was significantly inferior to that of the S-HBsAg (Joung et al., 2016). To overcome drawbacks of expressing M-HBsAg and L-HBsAg alone, original antigens with relevant, virus-neutralizing epitopes from the L protein inserted in the antigenic loop of S were successfully produced in *N. benthamiana* and *Lactuca sativa*. These antigens assembled in VLPs and proved more immunogenic than the S-HBsAg in vaccinated mice, validating this approach as a promising strategy for further development of improved HBV antigens (Dobrica et al., 2017; Pantazica et al., 2022).

In striking contrast to the focus on production of HBV envelope proteins in plants, the suitability of this system to express the HCV envelope proteins E1 and E2 has only recently been addressed. HCV-E2 is the main target of broadly neutralizing antibodies and the major candidate for the development of a vaccine (Bailey et al., 2019). E1 and E2 are heavily glycosylated transmembrane proteins that undergo complex processing and folding to achieve their native conformation and do not assemble in VLPs, tentatively explaining the difficulty to produce them in recombinant systems. The E1E2 dimer and an N-glycosylation mutant, E1E2ΔN6, were first produced in lettuce, using a transient gene expression system, and were found to be correctly processed and glycosylated. Oral vaccination with the antigen-synthesizing lettuce induced vaccine-stimulated secretion of IgA (Immunoglobulin A) in mice (Clarke et al., 2017). This study provided compelling evidence that this complex viral antigen could be produced, processed, and functionally modified in edible plants. A full-length E2 was also obtained in *N. benthamiana*, with a similar N-glycosylation pattern to that observed in mammalian cells (Dobrica et al., 2021). Parenteral administration of the purified antigen triggered a significant humoral immune response in mice and antibodies with HCV-neutralizing activity, despite the low dose used for immunization. Interestingly, expression of E2-derived epitopes as fusions to the B subunit of cholera toxin (CTB) in *N. benthamiana*, followed by mucosal administration of crude protein extract in mice, failed to generate a protective immune response, indicating the importance of epitope presentation in the appropriate conformation (Nemchinov et al., 2000).

Vaccination with HBV/HCV envelope proteins targets mainly the humoral arm of the immune system, resulting in production of virus-neutralizing antibodies that efficiently prevent infection, but are not able to eliminate the virus from infected cells. Interestingly, antibodies to the viral capsid (core) are found in large amounts in patients with chronic HBV/HCV infection, suggesting a role in viral clearance, which predestines this antigen for development of therapeutic vaccines. For this reason, low-cost production of both HBV and HCV core proteins was attempted in plants. Very high levels of HBV core (HBcAg) of several milligrams per gram fresh weight were obtained in transiently transfected *N. benthamiana*. The antigen self-assembled into capsid-like particles (which likely explains the non-toxic accumulation of large quantities in the plant leaves), and proved highly immunogenic in mice (Huang et al., 2006). The conjugation of tandem Hepatitis B core (tHBcAg) VLPs and the GFP antigen was achieved in *N. benthamiana* without altering VLP formation or GFP fluorescence by SpyTag/SpyCatcher system (Peyret et al., 2020). In the case of HCV, core-derived peptides, rather than the full-length protein, were successfully expressed in tobacco plants, either alone (Madesis et al., 2010) or fused to the HBsAg in frame with other HCV-derived epitopes (Mohammadzadeh et al., 2016).

While there is still a long road ahead before a plant-made HBV/HCV vaccine will make it to the clinic, the intense research efforts made in the past two decades have unequivocally established that plants provide the appropriate environment for production of high-quality, fully functional HBV/HCV vaccine candidates. Future studies should focus on increasing the antigen yields and optimizing methods for large-scale production to make the plant expression platforms more competitive.

### Ebola virus

The Ebola virus (EBOV) belongs to the family *Filoviridae*, which also includes a number of other highly pathogenic viruses like Marburg, Diablo, Stria and Thamno (Zampieri et al., 2007; Hoenen et al., 2019). EBOV was first reported in 1976, in the northern Democratic Republic of the Congo (Bowen et al., 1977). Six different species of Ebola viruses are currently known: Zaire, Sudan, Reston, Bundibugyo, Tai Forest and Bombali ebolavirus (Miranda and Miranda, 2011). EBOV infects human and nonhuman primates causing severe hemorrhagic fever. The virus enters through mucosal surfaces or skin scratches. It may cause acute death within 7-16 days (Ksiazek et al., 1999). The initial symptoms are non-specific flu-like symptoms such as fever, headache, muscle ache, malaise, later developing to vascular, neurological, respiratory and gastrointestinal distress (Zaki and Goldsmith, 1999). When the viral load increases to a certain level, multiorgan necrosis and coagulation disorders will set in (Bwaka et al., 1999). The Zaire ebolavirus is the most virulent species with a mortality rate of 25% to 90% (Beeching et al., 2014), and is classified as a category A pathogen (Cenciarelli et al., 2015).

Ebola is an enveloped virus and has a negative-strand RNA genome of 19 kb (Zampieri et al., 2007). Due to its high virulence, research on EBOV can only be conducted in contained facilities of biosafety level 4, limiting vaccine development and drug discovery. The nucleoside analogues favipiravir (T-705) and BCX-4430, and an immunomodulatory CpG oligodeoxynucleotide (ODN) have been established to be effective in EBOV treatment, but they are forbidden because of their high teratogenicity and other side effects (Warren et al., 2014; Sissoko et al., 2016). Z-Mapp (Mapp Biopharmaceuticals), a mix of three monoclonal antibodies, has been reported to effectively neutralize EBOV (Qiu et al., 2014).

Some non-replicative vector-based vaccines derived from the adenovirus 5 replicon (Sullivan et al., 2000), or the Venezuelan equine encephalitis virus (VEEV) replicon (Herbert et al., 2013), replicative vector-based vaccines (recombinant vesicular stomatitis virus-Zaire Ebola virus rVSV-ZEBOV) and antigen-based vaccines against EBOV are currently under clinical trials, but concerns remain regarding pre-existing immunity against the vectors used, virulence relapse, side effects in humans, cost-efficiency and shelf life in developing countries (Rosales-Mendoza and Salazar-González, 2014).

Plant-made vaccines based on expressed viral protein antigens of EBOV represent an attractive alternative to currently available drugs and vector-based vaccines. Successful expression of two epitopes of the EBOV glycoprotein (GP) as fusions to the B subunit of the *Escherichia coli* heat-labile enterotoxin (LTB) in tobacco promises to provide a cost-effective candidate vaccine against EBOV in the near future (Ríos-Huerta et al., 2017). Furthermore, an antigen-antibody fusion was developed in *N. benthamiana.* It uses a geminiviral replicon system to express an Ebola immune complex consisting of the Ebola virus glycoprotein GP1 fused to the heavy chain of humanized 6D8 IgG monoclonal antibody, which specifically binds to an epitope of GP1 (6D8 IgG-GP1 fusion protein). It was reported that immunization of mice with the purified immune complex resulted in the induction of anti-Ebola virus antibodies at levels comparable to those obtained with GP1 VLPs, conferring 80% protection from fatal EBV infection (Phoolcharoen et al., 2011). In addition to vaccines, the humanized and glycoengineered monoclonal antibody cocktail against Ebola GP (MB-003) is produced in *N. benthamiana* (ZMapp) and demonstrated a 43-100% survival of challenged rhesus macaques, depending on the treatment time after infection with EBOV. This cocktail of three antibodies is currently the most efficient drug against EBOV and was approved by the WHO for Ebola disease treatment (Qiu et al., 2014).

### Zika virus (ZIKV)

Zika virus belongs to the genus Flavivirus (family Flaviviridae) and occurs in two genotypes, an African and Asian variant (Faye et al., 2014). The virus is primarily transmitted through the mosquito *Aedes aegypti*, but also through the related species *A. africanus, A. albopictus* and *A. hensilli* (Gorshkov et al., 2019). Sexual contact, blood transfusions, and transmission from mother to fetus during pregnancy are additional possible modes of Zika infection (Gregory et al., 2017). Infected humans are either asymptomatic or develop fever and cutaneous rash (Paniz-Mondolfi et al., 2019). Fetal microcephaly results from vertical transmission and is highly associated with Zika virus (Antoniou et al., 2020). Therefore, the WHO declared Zika virus a “public health emergency of international concern” (de Araújo et al., 2016).

Like other Flaviviruses, Zika virus is enveloped, icosahedral and has a non-segmented, single-stranded, positive-sense RNA genome (of ~10 kb). Genetic similarity between strains can cause antibody-dependent enhancement of infection (ADE), posing an additional challenge to vaccine development (Valiant et al., 2018; Pierson and Diamond, 2020). Glycoprotein E with its DIII domain (zEDIII) is a principal candidate for development of a subunit vaccine due to its roles in virus attachment to cellular receptors, membrane binding and fusion for viral entry, its involvement in mediating viral assembly and its potential to induce potent neutralizing antibodies (Chambers and Monath, 2003; Wang et al., 2019). The ZIKV envelope protein E forms a heterodimer with another structural protein, precursor membrane protein (prM). When the two proteins were synthesized in *N. benthamiana* by transient expression, low protein accumulation and limited VLP formation were observed (Peyret et al., 2020). However, ZIKV E transiently expressed in *N. benthamiana* showed potent activation of E protein-specific antibody production and cellular immune responses in mice (Yang et al., 2018). When expressed in *N. benthamiana*, VLPs consisting of the HBV capsid protein HBcAg and presenting zEDIII also had the ability to trigger humoral and cellular immune responses in mice (Yang et al., 2017). zEDIII also could be fused to the IgG heavy chain to form recombinant immune complexes (N-RICs), which improved immunogenicity and led to up to 150 folds higher titers of Zika-specific antibodies in mice than administration of zEDIII alone (Diamos et al., 2020a). Moreover, joint administration of plant produced HBcAg VLP displaying ZE3 and RIC zEDIII RICs had a synergistic effect and triggered enhanced immune responses (Diamos et al., 2020b).

### Dengue virus (DENV)

Dengue virus (DENV), another flavivirus in the family Flaviviridae, is distributed in tropical and subtropical areas and transmitted by mosquitos (*A. aegypti* and *A. albopictus*). The WHO estimates that over 3.6 billion people are under risk of infection, and more than 200 million are infected, with 21,000 deaths occurring, every year (Murugesan and Manoharan, 2020). DENV infection may proceed asymptomatically, or result in self-limited dengue fever, which may progress to severe dengue haemorrhagic fever and dengue shock syndrome (Bäck and Lundkvist, 2013). The DENV genome has a single reading frame encoding three structural proteins (capsid protein, precursor membrane protein and envelope proteins) and at least seven non-structural (NS) proteins, all of which are produced from a single large precursor protein by proteolytic cleavage (Zhang et al., 2021).

The dengue DIII domain of the E protein (dEDIII) is considered as a primary target for vaccine development due to its potential to activate a neutralizing antibody responses (Leng et al., 2009; Chen et al., 2013). dEDIII and the premembrane protein (prM) have been successfully expressed in cowpea, lettuce chloroplasts, *N. benthamiana* and tobacco chloroplasts (Martínez et al., 2010; Kanagarajan et al, 2012; Carlos Marques et al., 2014; Gottschamel et al., 2016; van Eerde et al., 2019). For example, dEDIII was expressed in *N. tabacum* by stable nuclear transformation (Kim et al, 2015), chloroplast transformation (Gottschamel et al., 2016), or transient expression in *N. benthamiana*, with the latter approach leading to higher production levels (Chen et al., 2013; Kim et al., 2015; Gottschamel et al., 2016). dEDIII produced in lettuce chloroplasts for oral vaccination showed immunogenicity in rabbits (van Eerde et al., 2019). To display the antigen on the surface of HBcAg capsid-like particles (CLP), dEDIII was inserted into the immunodominant c/e1 loop region of tandem HBcAg, leading to the formation of chimeric VLPs in *N. benthamiana* (Saejung et al., 2007; Martínez et al., 2010). DENV VLPs were also produced by transient co-expression of DENV structural proteins, including the capsid (C), the precursor membrane (prM) and the envelope (E) protein, and a truncated version of the non-structural proteins (NS2B-NS3) in *N. benthamiana*. The immunogenicity was evaluated and high antibody responses in mice were found (Ponndorf et al., 2021). Finally, a chimeric Dengue-Ebola recombinant immune complex was developed with dEDIII fused to the heavy chain of the 6D8 monoclonal antibody (Dengue RIC technology, DERIC; Phoolcharoen et al, 2011). This approach led to enhanced anti-cEDIII immune responses, suggesting its promise for the development of a new candidate vaccine (Kim et al., 2015).

### Other zoonotic viruses

There have been many reports on attempts to develop plant-made vaccines against zoonotic viruses, including yellow fever virus (YFV), west Nile virus (WNV), rabies virus (RABV), Japanese encephalitis virus (JEV), hantaviruses (HVs), rift valley fever virus (RVFV), crimean-congo haemorrhagic fever virus (CCHFV), chikungunya virus (CHIKV), and hepatitis E virus (HEV) (**Table 2**). However, there are still many diseases caused by zoonotic viruses for which therapeutic options are completely lacking such as Marburg virus (MV), Hendra virus (HeV), Nipah virus (NiV), tick-borne encephalitis virus (TBEV) and others.

**Table 2:** Examples of plant produced antigens against zoonotic viruses.

Virus	Antigen	Plant platform	Immunogenicity	Reference
HIV	CTB-P1	*N. benthamiana*	Induction of mice serum IgG response, mucosal IgA response and immunological memory	Matoba et al., 2004
	p24, p24-Nef fusion	Tobacco	Strong serum IgG response, also with p24-Nef fusion protein as orally administered booster	Zhou et al., 2006; McCabe et al., 2008; Gonzalez-Rabade et al., 2011
	Tat	Tomato	Strong immune responses in mice through intraperitoneal, intramuscular or oral delivery.	Ramírez et al., 2007
	C4(V3)6	Lettuce	Induction of humoral and cell-mediated immune response in mice	Govea-Alonso et al., 2013
	Epitope from gp41	Cowpea	Strong serum neutralizing antibody response in mice	McLain et al., 1996; Porta et al., 1994; McLain et al., 1995
CCHFV	Gn/Gc glycoproteins	Tobacco	Induced IgG and IgA in orally administrated mice	Ghiasi et al., 2011
RVFV	Chimeric VLPc	*N. benthamiana*	Induced specific antibodies in mice	Mbewana et al., 2019
	N, Gn	*A. thaliana*	Showed immunogenicity in orally immunized mice	Kalbina et al., 2016
HV	N protein	Tobacco, potato	Induced specific IgG and IgA after oral or intraperitoneal administration in rabbits	Khattak et al., 2002
WNV	wEDIII	*N. benthamiana*	Induced protective immunity in mice	Lai et al., 2018; He et al., 2011; He et al., 2014
	wEDIII-HBcAg	*N. benthamiana*	B-cell and T-cell responses in mice	He J. et al., 2021
YFV	YFE, YFE-LicKM	*N. benthamiana*	Virus-neutralizing activity in mice, protection to over 70%	Tottey et al., 2017
JEV	E surface glycoprotein	Rice	Specific neutralizing antibodies in intraperitoneally injected mice and mucosal immune response in orally treated mice	Wang et al., 2009
	CVPs	*N. benthamiana*	Neutralizing antibodies in mice	Chen et al., 2017
RABV	G protein	Tobacco	Complete protection in mice	Ashraf et al., 2005
	N protein	Tomato	Partial protection upon parenteral immunization	Perea Arango et al., 2008
	G protein	Corn	Neutralizing antibodies, 100% protection	Loza-Rubio et al., 2012
	Epitopes from G and N fused with the AlMV coat protein	Tobacco, spinach	Specific response in human volunteers	Yusibov et al., 2002
HEV	Truncated 111N/54C	Potato	Absence of immune response in orally immunized mice	Zhou et al., 2006
	110-610 protein		Specific IgG antibodies in mice	Mardanova et al., 2020
	HBcAg-VLP		Induction of a specific antibody response	Zahmanova et al., 2021

## Plant-made vaccines against viral diseases in farm animals

### Avian influenza virus (AIV)

Avian influenza is caused by influenza A virus (IAV) belonging to the genus *Alphainfluenzavirus*, family *Orthomyxoviridae* (International Committee on Taxonomy of Viruses, ICTV). AIV particles are spherical, approximately 100 nm in diameter, or filamentous, about 300 nm in length, and contain an eighth-segmented, negative ssRNA genome (Palese, 2007). The glycoprotein spikes of haemagglutinin (HA) represent nearly 80% of the total surface proteins, neuraminidase (NA) represents 17%, and the ion channel matrix protein 2 (M2) is another major surface protein. The M1 protein surrounds the core virus particle. AIVs are divided into serotypes of 16 HA and 9 NA variants, giving rise to many different combinations (named, e.g., H5N1). During infection of the same cell with two or more serotypes, reassortment event (i.e., exchange of genomic segments) can take place and produce new serotypes (referred to as antigenic shift). Frequent appearance of point mutations (due to lack of proofreading activity of the genome-replicating RNA-dependent RNA polymerase) leads to antigenic drift, which may further contribute to escape from host immune responses (Harder et al., 2016). Low-pathogenicity avian influenza (LPAI) may cause mild to intense respiratory disease symptoms and a mortality of up to 20% in chickens (Pusch and Suarez, 2018). Highly pathogenic avian influenza (HPAI), often characterized by a polybasic cleavage site in HA (of variants H5 and H7), causes intense symptoms and can induce peracute death without clinical signs, thus leading to high mortality in chickens and turkeys (Swayne and Sims, 2021).

HPAI H5 and H7, and LPAI H9N2, have been widely used as prototypical antigens for plant made AIV vaccine preparation for application in poultry (Pusch and Suarez, 2018). There have been many attempts to express AIV antigens in a variety of plant species such as *N. benthamiana*, alfalfa, soybean, lettuce, *Arabidopsis thaliana*, *N. tabacum* and duckweed. For example, the H7 variant of the HA antigen was transiently expressed in *N. benthamiana* (Kanagarajan et al., 2012). Lettuce showed the lowest antigen production upon comparison of alfalfa, lettuce and soybean (Farsad et al., 2016). To improve protein accumulation, different signal peptides targeting the antigen protein to the ER, the apoplast or protein bodies were introduced and protein accumulation was compared in alfalfa. A signal peptide targeting the antigen to the apoplast yielded the highest protein accumulation level. Furthermore, full-length HA performed best when targeted to the apoplast, whereas a truncated HA form accumulated to higher levels in the ER (Mortimer et al., 2012).

In an alternative approach, the ectodomain of the H5 variant of HA was fused to the intrinsic trigger motif of GCN4 (general control transcription factor 4), which promotes protein folding, was expressed in *N. benthamiana*. Plant extracts containing HA oligomers induced higher titers of neutralizing antibodies than extracts containing HA trimers in immunized mice (Phan et al., 2020). Similar results have been obtained in chickens (Mokoena et al., 2019). HA can self-assemble to produce VLPs, but co-transformation of HA and M2 improved the efficiency of VLP production (Smith et al., 2020). Engineered *N. benthamania* with reduced xylosylated and/or core alpha1,3-fucosylated glycan structures made the *N*-glycosylation patterns more similar to that in vertebrates (Major et al., 2015). AIV VLPs formulated with the Montanide adjuvant elicited strong immune responses, and provided better protection against viral challenge in chickens. It was estimated that 1 kg of plant material is sufficient to provide vaccine for up to 30,000 chickens, suggesting high cost-efficiency of the plant-made antigen and suitability of the vaccine also for use in resource-poor areas. The HA variant H5 expressed in transgenic *A. thaliana* triggered robust immune responses and provided protection from lethal challenge with AIV when freeze-dried leaf powder was orally administrated in mice (Lee et al., 2015). Similarly, mice and ferrets immunized with a truncated H5 protein produced in *N. benthamiana* could be protected from AIV infection (Major et al., 2015).

The extracellular domain of M2 (of 23 amino acids) is highly conserved in all AIV variants, thus making vaccines developed from M2 potentially effective against all subtypes. The M2e peptide, when expressed in duckweed, provided protection in mice after immunization by oral administration (Firsov et al., 2018).

### Newcastle disease virus (NDV)

Newcastle disease virus (NDV) infects birds through oropharyngeal secretions and faecal matter, or inhalation of contaminated dust or aerosols containing the virus. There are four pathotypes that have been classified according to the disease severity they cause in chicken: asymptomatic enteric, lentogenic, mesogenic and velogenic. The strongest velogenic strains cause acute lethal infection, occasional haemorrhagic lesions in the intestine, and neurological and respiratory disorders leading to morbidity and mortality of up to 100% (Aldous and Alexander, 2001).

NDV is a paramyxovirus, also known as avian orthoavulavirus 1 (AOaV-1), that belongs to the genus Orthoavulavirus and the subfamily Avulavirinae. It is an enveloped virus with a non-segmented negative-sense RNA genome encoding six structural proteins: nucleoprotein (NP), phosphoprotein (P), matrix protein (M), fusion protein (F), haemagglutinin-neuraminidase (HN), and large polymerase (L). The HN, F and M proteins are related to the viral envelope. HN and F mediate viral entry and are the main targets of host neutralizing antibodies (Maclachlan et al., 2010).

Among these viral proteins, the glycoproteins F and HN have been expressed in various plants such as *N. benthamiana*, *N. tabacum*, *Zea mays*, *Solanum tuberosum* and *Oryza sativa*. The expression levels obtained suggested great potential for further development of these antigens as candidate NDV vaccine. The plant-produced glycoproteins were folded properly and correctly post-translationally modified (Margolin et al., 2020). As early as in 2006, recombinant NDV HN protein produced in transgenic tobacco-derived suspension cell cultures was found to give 90% protection against lethal NDV challenge in chickens after three doses of subcutaneous injection (Yusibov et al., 2011). NDV HN gene was also expressed in tobacco by transient expression. Six-week-old chickens fed with transgenic tobacco expressing HN of NDV with sterilized PBS did not show a specific immune response (Hahn et al., 2007). However, recombinant HN and F proteins expressed in transgenic potato could stimulate mucosal immune responses in mice upon oral immunization (Gomez et al., 2008). Transgenic corn expressing the F and HN proteins also induced production of locally secreted IgY (the avian IgG equivalent) in orally immunized chickens (Shahid et al., 2020).

Immune tolerance in poultry is much less common than in laboratory animals like rats and mice (Friedman, 2008). Therefore, application of transgenic plants as edible vaccines has also been explored for ND prevention. The NDV F protein expressed in staple crops like transgenic rice and corn was reported to induce the production of neutralizing antibodies in chickens challenged with a velogenic strain and provide similar survival rates as commercial LaSota vaccine based on the attenuated virus (Guerrero-Andrade et al., 2006; Ma et al., 2020). Recombinant concatenated F and HN proteins were expressed in transgenic tobacco but unfortunately induced unideal production of antibodies in rabbits (Shahriari et al., 2019).

### Nervous necrosis virus (NNV)

Over 40 farmed marine fish species are susceptible to viral nervous necrosis (VNN) caused by the nervous necrosis virus (NNV) (Lin et al., 2007; Walker and Winton, 2010). The disease is also called viral encephalopathy and retinopathy (VER), and its symptoms include skin darkening, abnormal swimming behaviour (Grotmol et al., 1997; Bovo et al., 1999; Thiery et al., 1999) and a mortality rate of up to 100% in larvae and early juvenile fish (Lin et al., 2007). NNV belongs to the betanodaviruses (Mori et al., 1992) in the family Nodaviridae. There are different genotypes of NNV such as striped jack NNV (SJNNV), tiger puffer NNV (TPNNV), barfin flounder NNV (BFNNV), redspotted grouper NNV (RGNNV) (Nishizawa et al., 1997), turbot NNV (TNNV) (Johansen et al., 2004) and three unclassified genotypes (Sahul Hameed et al., 2019). NNV particles are non-enveloped, and 25-30 nm in diameter. NNV contains two positive-sense RNAs, RNA1 encoding the RNA-dependent RNA polymerase and the non-structural protein B, and RNA2, encoding the coat protein precursor alpha, with RNA2 regulating RNA3 synthesis (Friesen and Rueckert, 1984; Mori et al., 1992).

The coat protein precursor can self-assemble into provirions (Gallagher and Rueckert, 1988; Cheng et al., 1994). NNV VLPs have the potential to be used for vaccine development. Dragon grouper NNV (DGNNV) VLPs and grouper NNV (GNNV) VLPs expressed in *E. coli* inhibited viral attachment to the surface of fish nerve cells and exhibited immune stimulatory activity in vaccinated fish (Lu et al., 2003; Lai et al., 2014), as well as stimulation of the complement system, an important component in teleost innate immunity (Lai et al., 2014). Plant-produced vaccines and especially VLPs, are potentially cost-efficient, which is particularly important in aquaculture (Marsian et al., 2019). The coat protein of Atlantic cod NNV (ACNNV) was expressed in *N. benthamiana* leaves and transgenic tobacco BY-2 cells, producing VLPs with similar structure to previously reported GNNV particles. The VLPs provided partial protection against ACNNV in intraperitoneally or intramuscularly immunized sea bass (*Dicentrarchus labrax*) (Marsian et al., 2019).

### Atlantic salmon piscine myocarditis virus (PMCV)

The cardiomyopathy syndrome (CMS) has been reported in both farmed and wild Atlantic salmon (*Salmo salar*) since the 1980s (Amin and Trasti, 1988; Poppe and Seierstad, 2003; Haugland et al., 2011) It leads to infiltration of mononuclear cells in the subendocardium, and degeneration and necrosis of the spongious myocardium (Bruno et al., 2013), which in turn can result in rupture of the atrium wall or sinus venosus and consequently sudden death (Bruno and Poppe, 1996). The causative pathogen, piscine myocarditis virus (PMCV), tentatively assigned to the Totiviridae family, is a small, non-enveloped icosahedral virus. Its single, linear double-stranded RNA genome encodes three open reading frames (ORF1-3), encoding a capsid protein and an RNA-dependent RNA polymerase. A subunit vaccine produced in plants has been established as an economical approach to combat PMCV infections (Clarke et al., 2017; van Eerde et al., 2019). There is currently no commercial vaccine available against PMCV, but the putative capsid protein of PMCV has the potential to be developed as an effective vaccine. The protein was transiently expressed in *N. benthamiana* and shown to form VLPs. The VLPs triggered innate immune responses, resulting in viral load inhibition in several tissues and reduction of lesions in the heart, as evaluated by a newly established PMCV infection model (Su et al., 2021). Plant-made VLP antigens against PMCV are worthy of further study towards providing protection from CMS in the future.

## Challenges and perspectives

Vaccines against viral diseases in humans and animals based on plant expression platforms have been developed in many plant species, including the grain crops rice and maize, and vegetable crops such as banana and tomato. Compared to traditional vaccines, the long shelf-life, the potential delivery as raw plant material, and the superior temperature stability (bypassing the necessity to maintain an uninterrupted cooling chain) are key attractions of plant-based edible vaccines (Yang et al., 2017). Although requiring more labour and longer time to produce, plant-made vaccines don’t have the strict limitations of instability and low temperature storage of mRNA-based vaccines. Nevertheless, plant made vaccines have a high requirement for costly infrastructure for downstream processing and purification as well as the necessary clinical trials. Compared to microbes and mammalian cells which require expensive fermenters and sterile conditions to ensure compliance with pharmaceutical Good Manufacturing Practice (GMP), plants cultivated in greenhouses or open fields are less expensive and easily scalable, but there are challenges in controlling quality and safety, as well as costly extraction and purification (Schillberg and Finnern, 2021). General factors that need to be addressed with plant-produced vaccines include batch-to-batch consistency, requirement for dosage standardization, dependence of product yields on environmental factors, plant development and plant health, and immunogenicity after downstream processing and (for oral vaccines) after food processing (e.g., cooking or drying) (Masavuli et al., 2017). Uncertainty in the business world is a further challenge, exemplified by the closure of Medicago (producer of the SARS-CoV-2 VLP vaccine) by its parent company, Mitsubishi.

Plant-based vaccines are subject to strict regulatory requirements, especially when produced in genetically modified plants. The use of transgenic technologies adds further issues that need to be dealt with, including biosafety concerns, people’s acceptance, the risk of contaminating the food chain, plant-specific post-translational modifications, and the possible development of oral tolerance to edible vaccines (Kirk et al., 2005). Nevertheless, proper regulatory measures, appropriate monitoring and risk management upon production and distribution should reduce these potential risks to an acceptable level.

To date, the successful application and potential for large-scale production of plant based VLP vaccines have been demonstrated for several viral diseases, including HBV, HPV, HIV and AIV. Furthermore, the plant-based production technologies have also been applied to antibody production for oral, topical or parenteral administration (Venkataraman et al., 2021; Monreal-Escalante et al., 2022).

Future research should contribute to overcoming the limitations and regulatory hurdles of plant-made viral vaccines such as low productivity, costs of downstream processing and slow translation to applications (reviewed by Schillberg and Finnern, 2021). To exploit the advantages of plant-produced viral vaccines and overcome their limitations will make plant molecular farming an important source of vaccines against viral diseases.

In summary, plant-made vaccines against viral diseases in humans or farm animals offer great potential in disease prevention and antiviral therapy. Many plant produced vaccines under clinical trials are likely to provide more evidence of safety and efficacy for future applications. It can be expected that the rapidly progressing field of plant-derived vaccines and biopharmaceuticals will be able to provide fast, safe, and cost-effective solutions for disease control and prevention, with substantial benefits especially to the developing world. As more and more products enter the market, a proper and, ideally, internationally harmonized regulatory framework for the production of recombinant vaccine proteins in (transgenic) plants should be developed and regularly reviewed to ensure that it remains fit for purpose.

## Author contributions

HS and JC initiated and designed the overall concept and wrote the manuscript. All authors contributed to the article and approved the submitted version.
